# Harnessing the Potential of mRNA Vaccines Against Infectious Diseases

**DOI:** 10.1111/1751-7915.70212

**Published:** 2025-08-25

**Authors:** Nouran Rezk, Siobhán McClean

**Affiliations:** ^1^ School of Biomolecular and Biomedical Science University College Dublin Dublin Ireland; ^2^ UCD Conway Institute of Biomolecular and Biomedical Research University College Dublin Dublin Ireland

**Keywords:** bacterial infection, clinical trials, dengue, influenza, malaria, mRNA vaccines, Mycobacteria, parasitic infection, *Pseudomonas aeruginosa*

## Abstract

mRNA vaccines have emerged as promising alternatives to conventional vaccines because of their flexible design, high immunogenicity, favourable safety profile, efficacy and potential for rapid clinical development. The accelerated development of mRNA vaccines during the COVID‐19 pandemic has revolutionised the field of vaccinology, highlighting their potential for combating emerging infectious diseases. The mRNA platforms can induce robust humoral as well as CD4+ and CD8+ T‐cell‐mediated immunity, offering broader protection than subunit protein vaccines. Consequently, they have been extensively studied against a wide range of viral, bacterial and parasitic infections, although the development of mRNA vaccines against bacterial and parasitic infections has lagged behind those targeting viruses. This review highlights recent studies on mRNA vaccine development and applications against a wide range of infectious diseases including non‐COVID viral infections, bacterial pathogens such as Mycobacteria or *Pseudomonas aeruginosa* and parasitic infections, including malaria. Moreover, it discusses key optimisation strategies and highlights candidates that have progressed to clinical trials, and the current challenges in enhancing immunogenicity and improving delivery systems.

## Graphical abstract

RNA vaccines are promising because of their flexible design, strong immunogenicity, safety and rapid development. Recent advances have enabled mRNA vaccines to target various pathogens, including viruses such as influenza and HIV, bacteria such as Mycobacteria and *Pseudomonas aeruginosa* , and parasites such as those causing malaria.

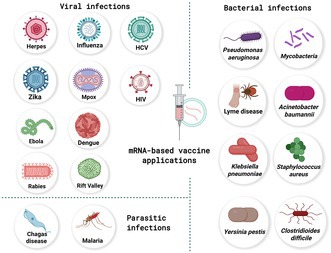

## 1 Introduction

**FIGURE 1:**
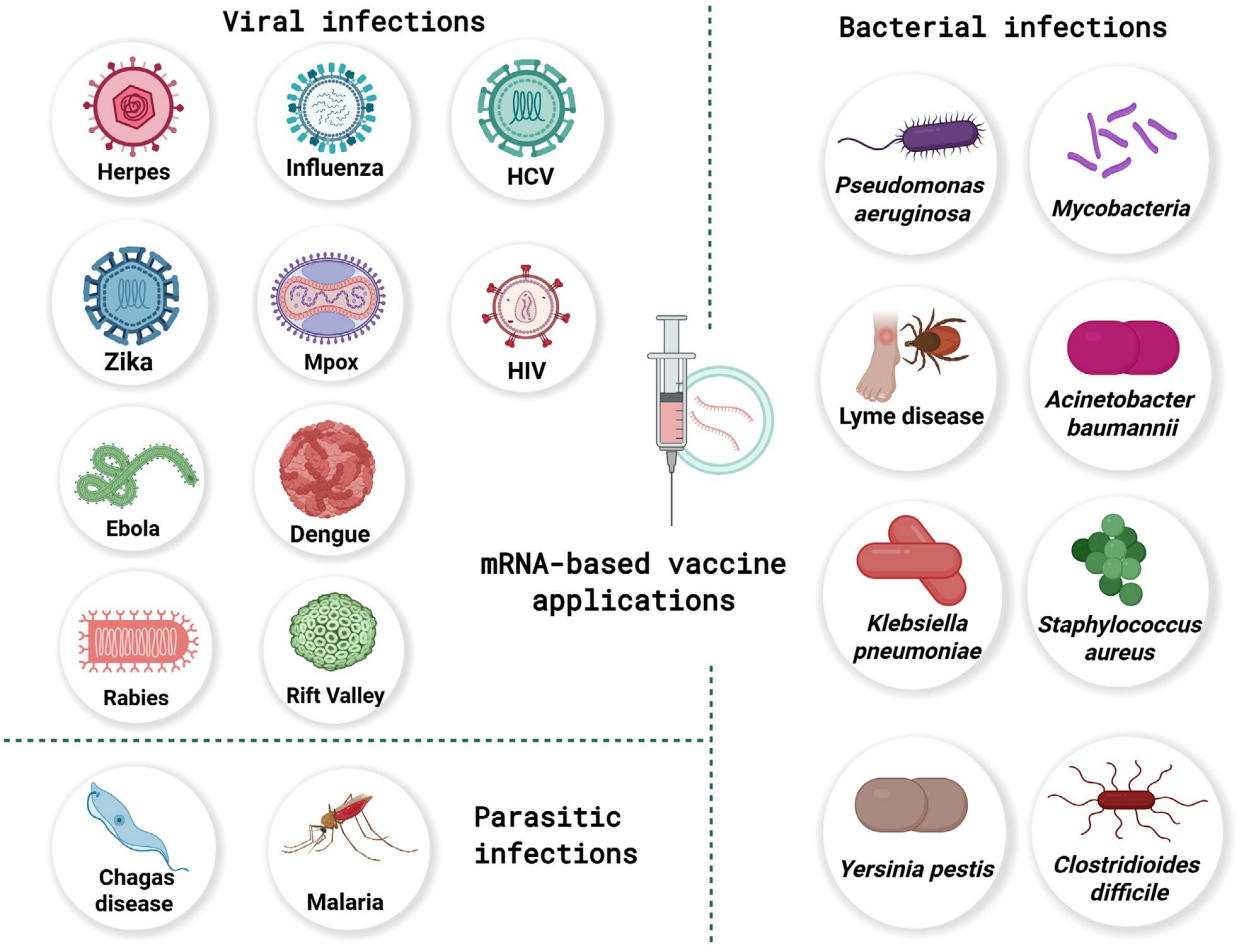
Examples of viruses, bacteria and parasitic infections that are being targeted with mRNA‐based vaccine platforms. Created with BioRender.com.

The development of mRNA vaccines represents a groundbreaking advancement in the field of vaccinology. They harness the body's cellular machinery to generate pathogen‐specific antigens, which, in turn, induce immune responses without direct exposure to the pathogen (Zhang, Tang, et al. 2023). By 2020, mRNA vaccines had been under investigation for several decades, although they had not yet achieved widespread clinical application (Verbeke et al. 2019). Before the COVID‐19 pandemic, the potential of mRNA therapeutic and prophylactic vaccines was widely recognised for infectious diseases including influenza, Zika virus, and rabies, in addition to cancer immunotherapy (Al Fayez et al. 2023), facilitating their rapid development and deployment in response to COVID‐19 (Pardi et al. 2018). The pandemic highlighted the potential of mRNA vaccines, with the Pfizer‐BioNTech and Moderna vaccines playing a crucial role in reducing viral transmission and the severity of infection, resulting in a reduction of up to 63% in global deaths during the first year of vaccination (Hogan and Pardi 2022; Watson et al. 2022). Consequently, the success of mRNA platforms has further accelerated interest in their evaluation against other infections (Chaudhary et al. 2021; Matarazzo and Bettencourt 2023). One of the main advantages of mRNA vaccines is that they can be customised and modified rapidly, which is particularly significant for pathogens that are constantly evolving (Dolgin 2021). Accordingly, they hold great promise in providing effective and flexible tools to combat both existing and future infectious diseases. To date, mRNA vaccines have been extensively evaluated for a wide range of viral infections, but their evaluation for bacterial or parasitic infections is relatively limited (Figure 1). We will review mRNA vaccines beyond COVID‐19 applications and will highlight their potential for other infectious diseases.

## 2 Types of mRNA Vaccines

**FIGURE 2:**
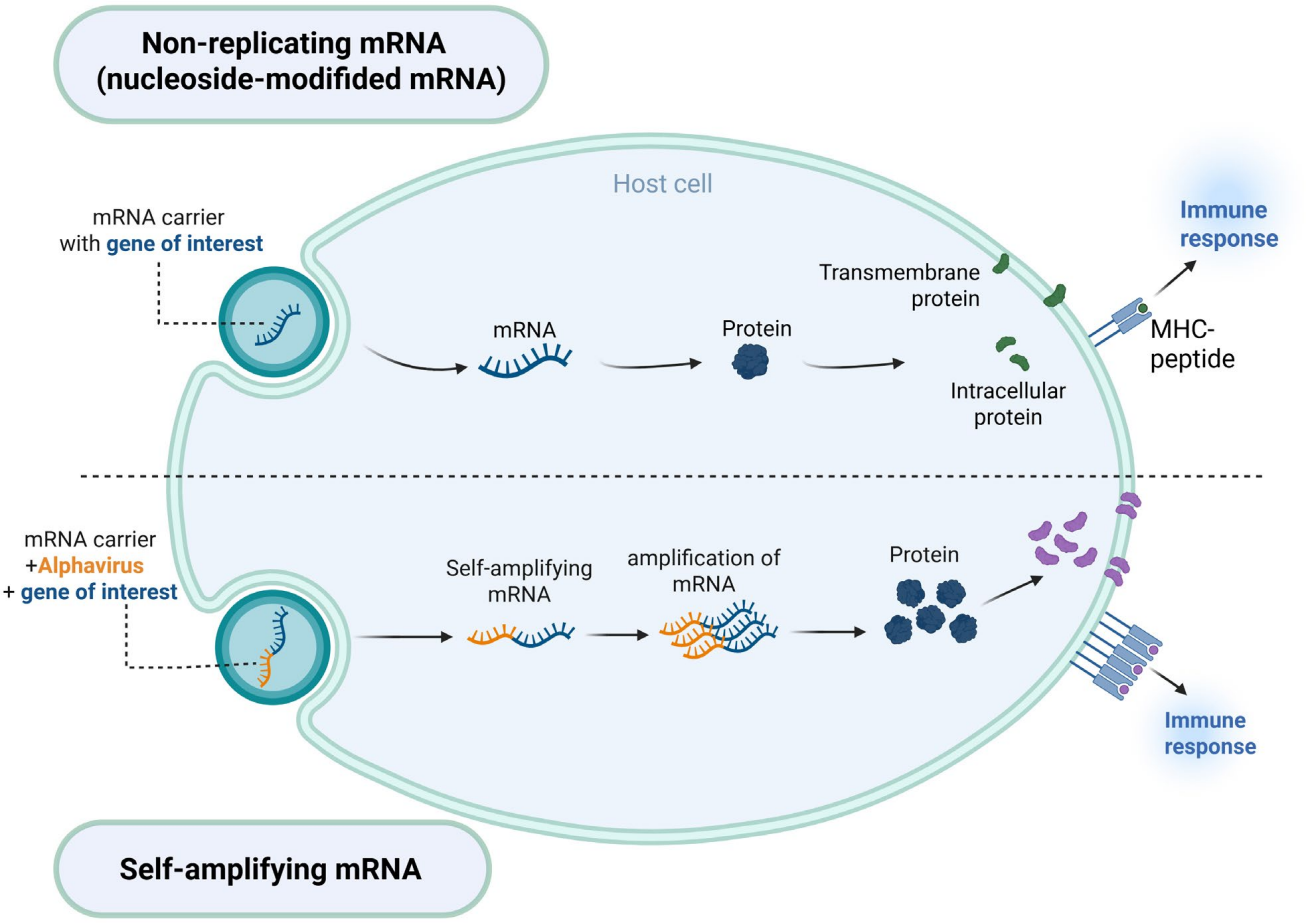
Schematic illustration of the key differences between non‐replicating mRNA and self‐amplifying mRNA vaccines within the host cell. Created with BioRender.com.

There are two main types of mRNA vaccines: non‐replicating mRNA vaccines and self‐amplifying mRNA vaccines (sa‐mRNA) (Figure 2). Non‐replicating mRNA vaccines are nucleoside‐modified (nm) that incorporate modified nucleosides, such as 1‐methylpseudouridine (m1Ψ), to evade recognition by RNA sensors, thereby reducing inflammation and enhancing antigen expression (Karikó et al. 2005, 2008). These vaccines are translated intracellularly into the target antigen without multiplication, minimising the risk of uncontrolled protein expression and associated side effects (Pardi et al. 2018). Alternatively, sa‐mRNA vaccines are designed with additional genetic elements that allow intracellular replication of the mRNA, resulting in robust and sustained protein expression (Bloom et al. 2021). Non‐replicating mRNA vaccines generally allow for a simpler manufacturing process and show a lower risk of reactogenicity, whereas sa‐mRNA vaccines may provide enhanced antigen expression and a longer duration of immune response. Recent research has revealed that at lower doses, sa‐mRNA demonstrates comparable immunogenicity to non‐replicating mRNAs, resulting in cost savings and increased accessibility (Gote et al. 2023).

## 3 mRNA Vaccines Targeting Influenza

Seasonal influenza viruses rapidly evolve with changes in antigenicity, particularly in hemagglutinin (HA), the main target of protective antibodies, resulting in the evasion of immunity (Becker et al. 2021). Therefore, it is no surprise that extensive research is being conducted on mRNA‐based vaccines targeting the variety of seasonal influenza strains (Table 1), and many candidates have proven more effective than conventional influenza‐based vaccines. A nm‐mRNA‐LNP vaccine expressing a wild‐type H3 antigen induced > 10‐fold more neutralising antibodies against the wild‐type 3c.2A H3N2 virus compared to both the conventional egg‐based vaccine adjuvanted with MF59 and an mRNA‐LNP encoding an egg‐adapted H3 antigen (Gouma et al. 2022). Two other HA‐mRNA vaccines, H10N8 and mRNA H7N9, were very effective in preclinical and phase 1 clinical trials, in which the vaccines showed favourable safety and high immunogenicity profiles, with 100% and 87% of patients having HAI titres ≥ 40 and microneutralisation (MN) titres ≥ 20 at day 43, respectively (Bahl et al. 2017; Feldman et al. 2019). Moreover, mice immunised with LNP‐mRNA vaccines encoding H1N1 HA elicited robust immune responses including high HAI titres, IgG, IgG1, and IgG2a antibodies, as well as IL‐4 and IFN‐γ‐producing CD4+ T cells (Zhuang et al. 2020). Importantly, this approach provided 100% survival against a lethal challenge and a 3‐fold reduction in viral loads in the lungs. A single 0.05 μg/antigen dose of a multivalent nm‐mRNA‐LNP vaccine encoding neuraminidase (NA), matrix protein 2 (M2), and nucleoprotein (NP) protected mice against seasonal influenza viruses, heterologous H1N1 strains (NC99 and PR8) and H5N8 and cH6/1 N5 (Freyn et al. 2020). The incorporation of signal peptides or ferritin (to exploit its nanoparticle potential) also conferred 100% survival against a H5N1 challenge (Zhuang et al. 2022). The Flu‐Fe/CD5‐Flu‐Fe vaccine incorporating ferritin exhibited more efficient expression of HA2 and M2e than its non‐ferritin counterpart, suggesting that mRNA vaccines combined with the self‐assembling nanoparticle potential of ferritin enhanced efficacy and provided robust protection.

**TABLE 1:** Examples of mRNA vaccines against non‐COVID viral infections.

Infectious agent	Antigen	Type^a^	Animal model	Route; Dose	Immune response	Bioburden/survival	References
Influenza	Wild‐type H3/3c.2A H3	nm	Mice	1 μg or 10 μg, i.m.	NAb	10‐fold reduction in viral load, 100% survival	Gouma et al. (2022)
	HA	nm	Mice, ferrets, NHP	0.4–10 μg (mice), 200 μg (ferrets)/0.2 and 0.4 mg (NHP), i.m. and i.d.	NAb, IFN‐γ	2 to 4‐log_10_ reduction in viral loads, 100% survival	Bahl et al. (2017)
	[H1/H3/H5/B‐HA2(aa90 ~ 105)‐M2e(24aa)], Flu‐Fe, and CD5‐Flu‐Fe with R848	nm	Mice	10 μg, i.m.	NAb, CD3 + CD4+ & CD3 + CD8+, high IL‐4, IL‐2, IFN‐γ	< LoD, 100% survival	Zhuang et al. (2022)
	HA + NA + M2 + NP + M1	nm	Mice	20 μg, i.d.	NAb, CD4+ T‐cell and CD8+ T‐cell (IFN‐γ, TNF‐α, and IL‐2)	100% survival	Freyn et al. (2021)
	HA + NA + M2 + NP	nm	Mice	5 μg/antigen or 1.25 μg/antigen	NAb, CD4+ T‐cell and CD8+ T‐cell (IFN‐γ, TNF‐α, and IL‐2)	100% survival	McMahon et al. (2022)
	HA + NA	nm	Mice	0.05–25 μg, i.d.	NAb, CD4+ and CD8+ T‐cells (IFN‐γ)	100% survival	Pardi et al. (2022)
	HA	nm	Mice, ferrets	50 μg (2.5 μg/antigen) mice)/60 μg (3 g/antigen) (ferret)	NAb	< LoD, 100% survival	Arevalo et al. (2022))
	H5 and N1	sa	Mice, ferrets	0.01–1 μg (mice)/0.5–5 μg (ferret), i.m.	NAb, CD4+ T‐cell (IFN‐γ, TNF‐α, and IL‐2)	< LoD	Chang et al. (2022)
RABV	RABV‐G	nm	Mice, dogs	0.2–5 μg (mice)/5 or 25 μg (dogs), i.m.	NAb, IgG	100% survival	Li et al. (2022)
	CpG 1018, CpG 2395 and Poly I:C	nm	Mice	10 μg, i.m.	NAb, CD4+ and CD8+ T cells (IFN‐γ and TNFα)	100% for pre‐exposure, 75% post‐exposure	Hongtu et al. (2023)
	Codon‐optimised GP	nm	Mice	0.3–10 μg, i.m.	NAb, IgG2a, IFN‐γ	15.2‐fold reduction in viral loads, 100% survival	Bai et al. (2023)
RVF	GP	nm	Mice, NHP	5 μg (mice)/100 μg (NHPs), i.m.	NAb, IgG, CD8+ T‐cells (IFN‐γ and TNF‐α) in mice/IFN‐γ, IL‐2 and IL‐4 and memory B cell in monkeys	< LoD,100% survival	Bian et al. (2023)
Ebola Virus	GP + NP	sa	Mice	2.5–7.5 μg saRNA (i.m. and i.d.)	NAb, IgG, CD4 + ‐specific T cell and CD8 + ‐specific T cell (IFN‐γ)	< LoD,100% survival	Krähling et al. (2023)
Crimean‐Congo haemorrhagic fever	Non‐optimised small (S) segment	mRNA	Mice	25 μg, i.m.	NAb, IgG1, IgG2a, IL‐4, IFN‐γ	100% survival	Farzani et al. (2019)
	GP + NP	nm	Mice	20 μg, i.d.	NAb and IFN‐γ	3‐fold reduction in viral loads, 100% survival	Appelberg et al. (2022)
	GP + NP	sa	Mice	2.5 μg of each RNA, or 5 μg total RNA for repNP + repGPC, i.m.	IgG, IFN‐γ	> 2‐log_10_ reduction in viral loads, 100% survival	Leventhal et al. (2022)
Zika virus	prM‐E	nm	Mice, NHP	3 and 0.6 μg (mice) and 10–200‐μg in (NHPS), i.m.	NAb	< LoD	Bollman et al. (2023)
	prM‐E	nm	Mice	5 μg, i.m.	IgG1, IgG2a, CD4+ and CD8+ T‐cell (IFN‐γ, TNF‐α)	6‐log_10_ reduction, 100% survival	Medina‐Magües et al. (2021)
	prM‐E	sa	Mice	1 μg, i.d.	NAb, IgG, CD4+ and CD8+ (IFN‐γ)	< LoD, 100% survival	Zhong et al. (2019)
	Domain III of prmE	nm	Mice	30 μg, i.m.	NAb, IgG1, IgG2c, IFN‐γ, TNF‐α, IL‐2 and IL‐6	1‐log_10_ reduction	Lee et al. (2025)
HIV	Env and Gag proteins	nm	Mice, monkeys	2.5 μg, i.m.	NAb, CD4+ (IFN‐γ, IL‐4, IL‐21)	N/A	Zhang et al. (2021)
	HIV‐1 Gag	nm	NHP	25 and 100 μg, i.m.	Anti‐p24Gag antibodies, low CD4+ (IFN‐γ)	N/A	Valentin et al. (2022)
	tHIVconsv1 and tHIVconsv2	sa	Mice	5 μg, i.m.	CD4+, CD8+ T‐cell (IFN‐γ)	N/A	Moyo et al. (2019)
	Native‐like HIV‐1 Env trimers	sa	Mice, guinea pig, rabbit, NHP	Mice (0.1 and 10 μg)/guinea pigs (8 μg) and rabbits (16 μg)	IgG1 and IgG2a, IFN‐γ	N/A	Aldon et al. (2021)
DENV	prME, E80and NS1	nm	Mice	5–10 μg, i.m.	NAb, IgG, IFN‐γ	< LoD	Zhang et al. (2020)
	prM and Env	nm	Mice	3–10 μg, i.m.	NAb, IgG, CD4+ and CD8+ T‐cell (IFN‐γ)	100% survival	Wollner et al. (2021)
	DENV1‐NS	nm	Mice	10 or 2 μg, i.m.	NAb, IgG2a CD8+ T‐cells (IFN‐γ and TNF‐α)	< LoD	Roth et al. (2019)
	E‐DIII and NS1	nm	Mice	2, 5 and 20 μg, i.m.	NAb, IgG, IFN‐γ	< LoD	He et al. (2022)
HCV	ectodomains of E1, and E2	m	Mice	10 μg, i.m.	NAb, IgG, IL‐2 and IFN‐γ	< LoD	Patra et al. (2023)
Chikungunya virus	E1 + E2	nm	Mice	10 μg, sc	NAb, and IFN‐y CD8+ T‐cell	N/A	Ge et al. (2022)
HSV	HSV‐2 gC2, gD2, and gE2	nm	Mice, guinea pigs	10 μg (mice)/20 μg (guinea pigs), i.m. and i.d.	NAb, IgG, CD4+ T‐cells	< LoD,100% survival	Awasthi et al. (2019)
	gC2, gD2, and gE2	nm	Mice, guinea pigs	20 μg each (total 60 μg), or 3 doses 10 μg each (30 μg total), i.m. and i.d.	NAb, IgG	100% survival	Awasthi et al. (2021)
	Ectodomains of the gC1 (27–457), gD1 (26–333), and gE1 (24–309)	nm	Mice	1 μg or 10 μg (total), i.m.	NAb, CD8+ T‐cells (IFN‐γ, TNF‐α, and IL‐2)	3‐log_10_ reduction in viral load, 100% survival	Egan et al. (2023)
M.pox	A29L, M1R, A35R and B6R	nm	Mice	40 μg, i.m.	NAb, IgG and CD8+ Tem cells	< LoD	Sang et al. (2023)
	M1R, E8L, A29L, A35R, and B6R	nm	Mice	5 μg, i.m.	NAb, IgG, CD4+ (IFN‐γ, IL‐2, and TNF‐α)	< LoD	Zhang, Tang, et al. (2023); Zhang, Wang, et al. (2023)
	M1, H3, A29, E8, B6 and A35	nm	Mice	1 μg or 5 μg, i.m.	NAb, IgG, CD4+ (IFN‐γ, and IL‐2)	100% survival	Zeng et al. (2023)

Abbreviations: < LoD, below the limit of detection; NAb, neutralising antibodies.

^a^ Type of mRNA (nucleoside‐modified; self‐amplifying).

There are ongoing efforts to develop multivalent mRNA vaccines to address a broader spectrum of influenza virus strains (Cheng et al. 2024). Unmodified mRNA‐LNP vaccines encoding Cal09 HA and Sing16 HA or Sing16 NA, Mich15 NA were evaluated as monovalent and multivalent formulations against seasonal and pandemic influenza (Chivukula et al. 2021). Both formulations elicited strong HAI titres and provided nearly complete protection against lethal challenge in mice, and immunised NHPs developed IFN‐γ‐secreting cells and low IL‐13 levels, indicating a Th1‐type immune response. Co‐delivery of multiple mRNA transcripts encoding two or four antigens induced antibody titres comparable to those of individually formulated LNPs, with no signs of immunodominance or antigen interference. Other combinations of multivalent nm‐mRNA‐LNP vaccines have also resulted in 100% survival against a broad range of influenza viruses (McMahon et al. 2022; Kackos et al. 2023), with some being 100% protective at a 0.05 μg dose per antigen against a lethal challenge (Table 1; Pardi et al. 2022). Furthermore, a multivalent nm‐mRNA‐LNP vaccine encoding HA from all 20 known influenza A virus subtypes and influenza B virus lineages elicited high levels of cross‐reactive and subtype‐specific antibodies including neutralising antibodies against H1N1, H3N2, H5N1, and H7N9, in mice and ferrets, resulting in 100% survival and undetectable viral loads in the lungs (Arevalo et al. 2022). In a Phase 1/2 clinical trial, a quadrivalent mRNA vaccine, mRNA‐1010, encoding HA from four influenza strains, was well tolerated and induced HAI titres reaching 1:160 across all strains, exceeding the 1:40 threshold associated with 50% protection (Lee et al. 2023). Moreover, the vaccine also induced durable HAI titres that persisted up to 6 months, along with high T‐cell responses (Ananworanich et al. 2025). Consequently, the vaccine progressed to Phase 3 and showed a favourable safety profile with high HAI titres (Kandinov et al. 2025, 3). At a low dose (0.01 μg), a sa‐mRNA encoding HA and NA antigens from H5N1, H3N2, H1N1 and Yamagata influenza strains elicited robust neutralising antibodies and CD4+ and CD8+ T‐cell responses in mice and protected ferrets from influenza at a 0.5 μg dose, with undetectable viral loads (Chang et al. 2022).

## 4 mRNA Vaccines Targeting Other Negative‐Sense RNA Viruses

Several other negative‐sense RNA viruses have also been targeted by mRNA vaccines. A CV7202 mRNA‐LNP vaccine encoding glycoprotein (RABV‐G) was developed against Rabies Virus (RABV), and the preliminary findings of a Phase 1 trial revealed all doses were well tolerated and elicited rabies‐neutralising antibodies meeting WHO protection criteria (Aldrich et al. 2021). However, the 5‐μg dose showed unacceptable reactogenicity. A more refined mRNA vaccine, LVRNA001, expressing RABV‐G with increased GC content and a poly(A) tail with a length of 100A, demonstrated a safety profile in mice and dogs (Li et al. 2022). It induced neutralising antibody levels exceeding the protective threshold (> 0.5 IU/mL) in mice and maintained high IgG antibody titres for up to 6 months. A robust IFN‐γ immune response was observed, and all the immunised mice survived the lethal challenge. In dogs, the vaccine elicited higher neutralising responses than the control group. It resulted in 100% survival over 3 months in rats, compared to Rabvac controls, which showed only 33% survival. An unmodified mRNA‐LNPs vaccine elicited neutralising titres in all immunised NHPs but one, above the WHO‐recommended threshold and superior to the Rabipur group (licensed vaccine; Hellgren et al. 2023). By week 18, all boosted groups exceeded the WHO threshold; however, only the boosted mRNA group sustained titres above this level in all animals over the 50‐week study. The mRNA vaccine induced high CD4+ Th1 responses, in contrast to the Rabipur group; however, CD8+ T‐cell responses were undetectable in all groups. Two mRNA vaccines, RV021 and RV022, encoding the RABV‐G of the CTN‐1 strain with a standard or shorter poly‐A tail, respectively, were compared in mice (Li et al. 2023). Serum‐neutralising antibody titres were higher in the RV021 group and sustained for at least 260 days, stimulated a robust IFN‐γ immune response, and 100% survival was achieved at a 1 μg dose. A RABV‐G‐mRNA‐LNP combined with CpG 1018 elicited the strong, stable antibody responses and higher neutralising titres for up to 60 weeks than non‐CpG 1018 groups (Hongtu et al. 2023). It also induced higher frequencies of antigen‐specific CD4^+^ and CD8^+^ T cells and conferred 100% survival in mice. A single‐dose nm‐mRNA‐LNP encoding a codon‐optimised G protein elicited high binding titres and virus‐neutralising activity five‐fold higher than those observed in inactivated vaccine groups and Th1‐biased responses (Bai et al. 2023). Remarkably, single RABV‐G mRNA immunisation, even at low doses, achieved 100% survival against lethal rabies challenges and durable humoral immune responses for at least 25 weeks, whereas a two‐dose regimen extended the protective response for 1 year. In a post‐exposure model with lethal challenge before immunisation, a RABV‐G mRNA vaccine, SYS6008, exhibited survival rates of 77%–94.3%, surpassing the commercial vaccine at the same dose (Yu et al. 2023). Other nm‐mRNA vaccines encoding wild‐type G, soluble trimeric G (tG‐MTQ), membrane‐anchored prefusion‐stabilised G (preG), VLP formulations encoding preG and M to generate VLPs and VLP/N mRNA vaccine co‐expressing preG, M and N were developed (Liu et al. 2024). The preG, VLP, and VLP/N mRNA vaccines induced IgG titres 2.4‐, 3.5‐, and 2.1‐fold higher than the wild‐type G vaccine, with a Th1‐biased response. All exceeded the protective neutralising antibody threshold, with preG, VLP and VLP/N inducing titres 1.9‐, 1.7‐ and 1.7‐fold higher than G, respectively. They also elicited stronger IFN‐γ + CD4+ T‐cell responses than the licensed vaccine. All candidates conferred 100% survival in immunised mice.

Six nm‐mRNA constructs encoding varying lengths of the Rift Valley Fever (RVF) Gn and Gc proteins elicited 50% focus reduction neutralisation test (FRNT_50_) titres 1000‐fold greater than the control group, indicating a strong neutralising antibody response (Bian et al. 2023). It also induced high levels of antigen‐specific IgG and enhanced CD8+ T‐cell responses. The mRNA‐GnGc demonstrated 100% survival and undetectable viral loads in the spleen and liver after the challenge in IFNAR−/− mice. Similarly, mRNA‐GnGc induced high levels of neutralising antibodies and cellular responses, and antigen‐specific memory B‐cells. Two sa‐mRNA vaccines encoding either WT consensus Gn and Gc or the Furin‐T2A sequence induced dose‐dependent RVFV Gn‐IgG antibody responses, which significantly increased after boosting (Kitandwe et al. 2024). Both formulations generated similar antibody levels; however, the WT consensus vaccine elicited the highest RVFV pseudovirus‐neutralising activity (IC_50_ = 551), followed by the 1‐μg dose (IC_50_ = 507). No neutralising activity was detected in unimmunised mice or low doses of the furin‐T2A vaccine.

There is an approved Ebola live attenuated vaccine (VSVΔG‐ZEBOV‐GP, ERVEBO), which has some limitations including the potential for reversion to a virulent form and instability under various storage conditions (Lee et al. 2024). Therefore, there is a need for safer and more stable vaccine alternatives (Malik et al. 2023). An EBOV‐saRNA‐LNPs vaccine expressing EBOV‐GP, either alone or in combination with the NP, induced high antibody and cellular responses (IFN‐y; Krähling et al. 2023). Following EBOV challenge in IFNAR−/− mice, no detectable virus in the serum, liver, or spleen of immunised groups was observed; and 100% survival following a lethal challenge was achieved.

In the case of Crimean‐Congo Hemorrhagic fever, a naked mRNA vaccine expressing the non‐optimised small segment of the Ank‐2 strain of the CCHFV exhibited 100% protection after a two‐dose regimen (Farzani et al. 2019). However, no neutralising activity was observed following either single or booster doses. An nm‐mRNA‐LNP encoding CCHFV NP or GcGn elicited robust humoral and IFN‐γ responses in both IFNAR−/− and immunocompetent mice and protected IFNAR−/− mice from lethal CCHFV infection (Appelberg et al. 2022). A sa‐mRNA vaccine, expressing either NP alone or with GcGn of CCHFV, also showed 100% survival in C57BL6 mice (Leventhal et al. 2022). Notably, a single immunisation with 100 ng of mRNA was effective in providing immunity against a lethal challenge.

## 5 mRNA Vaccines Targeting Positive‐Sense RNA Viruses

Since the 2015 Zika virus (ZIKV) outbreak raised global health concerns, efforts to develop effective vaccines have accelerated (Mittal et al. 2017). Moderna initially developed mRNA‐1325 on the basis of the prME ORF from a Micronesia 2007 ZIKV isolate, and later mRNA‐1893 from the RIO‐U1 strain (Richner et al. 2017; Bollman et al. 2023). In NHPs, mRNA‐1893 immunisation led to undetectable viral loads following challenge. The enhanced immunogenicity of mRNA‐1893 was linked to a single amino acid residue difference in the E protein ORF, evidenced by introducing an Ala40Thr substitution into mRNA‐1325, which restored virus‐like particle (VLP) secretion and immunogenicity to levels comparable to mRNA‐1893. Consequently, mRNA‐1893 advanced into phase 1 clinical trials, and was well tolerated and induced robust neutralising and binding antibody responses that persisted for up to 1 year (Essink et al. 2023). Subsequently, mRNA‐1893 progressed to Phase 2 in healthy participants that were either flavivirus‐seronegative or seropositive (ModernaTX Inc. 2024). A sa‐mRNA‐prmE vaccine exhibited a 10‐fold increase in antibody titres and 100% seroconversion in IFNAR1−/− mice; however, 25% of BALB/c mice failed to develop detectable antibody titres, and only 50% seroconversion was observed in C57BL/6 mice, highlighting the inhibitory effect of type I IFN on antibody responses (Zhong et al. 2019). Following a challenge in IFNAR1−/− mice, the vaccine provided complete protection with 100% survival, compared to 43% in controls.

Despite decades of research, no HIV‐1 vaccine candidate has yet succeeded in eliciting broadly neutralising antibodies in humans (Stephenson et al. 2020). Therefore, mRNA vaccines are a promising platform in addressing the high mutation rate of HIV (Ahmed and Herschhorn 2024). An nm‐mRNA vaccine, encoding Env and SIV Gag to form VLPs, induced neutralising antibodies in mice and NHPs (Zhang et al. 2021). Although all NHP controls were infected after 3 weeks, 29% of immunised NHPs remained uninfected after 13 weekly challenges and 71% of immunised NHPs showed delayed infection with significantly prolonged virus‐free survival. However, a regimen involving 10 doses will present challenges in humans, and further optimisation is required. Two mRNA HIV‐1 vaccine candidates, using either unmodified vectors or nm vectors encoding a T‐cell multiepitope construct (TEMP), were compared (Gómez et al. 2021). The unmodified mRNA induced low levels of CD8+ T‐cells, which were significantly enhanced by a booster dose with a poxvirus MVA‐TMEP vector expressing the same multiepitope protein in immunised mice. This prime/boost strategy led to a substantial increase in T‐cell responses, primarily the CD8+ T‐cell compartment, which was sustained for 3 months. An mRNA vaccine targeting conserved HIV Gag regions induced strong antibody responses but weaker T‐cell responses than a DNA vaccine; combining both vaccines in a prime/boost regimen enhanced cellular and humoral immunity (Valentin et al. 2022). In contrast, a single dose of a bivalent‐mosaic immunogen tHIVconsvX sa‐mRNA elicited strong T‐cell responses that remained stable for up to 22 weeks post‐immunisation (Moyo et al. 2019). Homologous sa‐mRNA boosts induced balanced CD8+ and CD4+ T‐cell responses, whereas the strongest T‐cell responses were observed when sa‐mRNA priming was combined with a heterologous viral vector boost, highlighting the importance of optimising T‐cell responses for HIV‐1 control. There is an ongoing phase 1 clinical trial for evaluating the safety and immunogenicity of eOD‐GT8 60mer mRNA Vaccine (mRNA‐1644) and Core‐g28v2 60mer mRNA Vaccine (mRNA‐1644v2‐Core; Clinicaltrials.gov 2021). Preliminary data showed that the mRNA‐1644 induced VRC01‐class IgG B‐cells in 97% of participants, and polyfunctional helper T‐cell responses were also induced (Venkatesan 2021; Cohen et al. 2023). However, because VRC01 antibodies require further mutation into broadly neutralising antibodies, the vaccine's efficacy remains unknown. Another phase 1 clinical trial on three mRNA vaccines (BG505 MD39.3, BG505 MD39.3 gp151 and BG505 MD39.3 gp151 CD4KO HIV Trimer mRNA Vaccines) is ongoing (NCT05217641).

In regard to dengue virus (DENV), three mRNA‐LNP vaccines, encoding prME, E80 and NS1 from DENV‐2, demonstrated immunogenicity to date, particularly E80‐mRNA alone or in combination with NS1‐mRNA, with high levels of neutralising antibodies and complete protection against DENV‐2‐GZ‐LP challenge (Zhang et al. 2020). However, E80‐mRNA+NS1‐mRNA elicited a lower titre of NS1‐specific antibody than NS1‐mRNA alone, suggesting an antigenic competition between the two proteins. Mice immunised with E80‐mRNA and E80‐mRNA+NS1‐mRNA had no measurable viremia 4 days post‐challenge. Similar results were observed with an nm‐mRNA‐LNP vaccine encoding prM and E from DENV1 that demonstrated both humoral and IFN‐γ immune responses (Wollner et al. 2021). Following a lethal challenge, 100% of DENV1 prM/E mRNA‐LNP‐immunised mice survived to day 32 post‐challenge. A nm mRNA‐LNP vaccine was developed to express DENV1‐NS, containing conserved and highly antigenic epitopes from NS3, NS4B and NS5 regions of DENV1, a shared sequence found in various DENV serotypes (Roth et al. 2019). Immunisation in human HLA class I transgenic mice, with type I IFN receptor blockade, revealed strong induction of IFN‐γ and TNF‐α CD8+ T‐cells. Following a challenge, 83% of immunised mice showed no viremia, whereas 80% of control mice developed viremia. A multivalent mRNA‐LNP vaccine encoding E‐DIII and NS1 derived from diverse viral strains elicited neutralising antibodies against DENV 1–4 strains and a Th1‐biased response including high IgG2a titres and IFN‐γ levels (He et al. 2022).

Hepatitis C virus vaccine development has been challenging because of limited animal models, the diverse nature of HCV genotypes, limited knowledge about the protective immune response, and the choice of a viral antigen for optimal protection (Duncan et al. 2020). However, mRNA‐LNPs encoding individual ectodomains of E1, E2 or a modified E2 (with reduced CD81 binding and an inserted N‐linked glycosylation site) were investigated (Patra et al. 2023). The sE2F442NYT nm mRNA‐LNP vaccine elicited a four‐fold increase in antibody binding and protective cellular responses compared to the unmodified vaccine; it conferred complete protection with no detectable viral titre post‐challenge.

There have been limited mRNA‐based vaccines against a range of other positive strand RNA viruses. In the case of Yellow Fever (YF), only two candidates (encoding the YFV prM‐E and NS1) have been reported. These induced robust humoral immune responses, resulting in complete protection against lethal YF virus infection after passive administration of serum or splenocytes from immunised mice (Medina‐Magües et al. 2023). Furthermore, immunised NHPs showed elevated expression of IFN‐α and IL‐1RA cytokines and I‐TAC and MCP‐1 chemokines, indicating a Th1‐biased immune response. Both vaccines also induced sustained high humoral and cellular immune responses for at least 5 months. There is no vaccine available to prevent Chikungunya infection. The CHIKV E2‐E1 mRNA vaccine was developed and showed robust neutralising antibody and IFN‐γ and IL‐4 CD8+ T‐cell responses, surpassing the subunit vaccine (Ge et al. 2022). In addition, mRNA‐1388 by Moderna was evaluated in a phase 1 clinical trial and showed a safety profile with 100% seroconversion and long‐lasting neutralising antibody responses in healthy adults for 1 year (Shaw et al. 2023).

## 6 mRNA Vaccines Against DNA Viruses

mRNA vaccines targeting infections caused by DNA viruses, such as HSV‐1 and HSV‐2, the causative agents of genital herpes, are also showing promising results (Dropulic and Cohen 2012). A trivalent nm‐mRNA‐LNP vaccine, encoding glycoproteins C, D, and E from HSV‐2, showed superiority over the trivalent subunit vaccine adjuvanted with CpG/alum, achieving sterilising immunity in 98% of mice, compared to 77% in the protein group (Awasthi et al. 2019). A significant reduction in viral shedding was also observed following a lethal HSV‐2 challenge in guinea pigs. A subsequent study showed HSV‐2 trivalent mRNA‐LNPs resulted in 100% survival with no genital disease in mice challenged with both HSV‐1 and HSV‐2 and hindered HSV DNA from reaching the dorsal root ganglia, the site of virus latency (Egan et al. 2020). Furthermore, the vaccine induced antigen‐specific memory B‐cells persisting for up to 1 year, highlighting durable immune responses (Awasthi et al. 2021). Notably, mice challenged 8 months after immunisation showed more durable protection in the mRNA group than the protein group. A trivalent HSV‐1 mRNA LNP vaccine, comprising gC1, gD1 and gE1, showed comparable HSV‐1 neutralising antibody titres to the trivalent HSV‐2 vaccine in a murine genital infection model, whereas the HSV‐2 vaccine induced more robust CD8+ T‐cell responses to gE1 peptides than the HSV‐1 vaccine (Egan et al. 2023). Both vaccines demonstrated complete protection against clinical disease in a lip model and prevented mortality and genital disease in a genital model. Consequently, the tri‐HSV‐2 vaccine is currently undergoing a phase 1 clinical trial (NCT05432583).

Another DNA virus, Monkeypox (MPV), was targeted by three mRNA vaccines encoding the A35R extracellular domain fused to M1R (VGPox1 and VGPox2), and a mixture of encapsulated full‐length A35R and M1R (VGPox3) (Hou et al. 2023). All vaccines elicited early anti‐A35R antibodies, but only VGPox1 and VGPox2 showed detectable levels of anti‐M1R antibodies. Although all mRNA vaccines demonstrated partial to nearly complete neutralisation of the virus at a 1:50 dilution, VGPox1 and VGPox2 exhibited significantly higher neutralising capacity than VGPox3 at a 1:500 dilution. At a single dose, all vaccines conferred 100% survival, with durable immunity and protection sustained up to day 162 post‐immunisation. Penta‐ or quadri‐valent vaccines encoding M1R, E8L, A29L, A35R and B6R significantly elicited neutralising antibodies in immunised mice with titres of 1:13,383 (AR‐MPXV5) and 1:8156 (AR‐MPXV4a; Zhang, Wang, et al. 2023). They also elicited antigen‐specific CD4+ T‐cells expressing Th1 cytokines (IFN‐γ, IL‐2, and TNF‐α) and protected the mice against VACV challenge. Two other quadrivalent mRNA vaccines elicited high IgG titres against all four MPXV antigens and increased neutralising antibody titres by ~2 log_10_, with a Th1‐biased response in immunised mice (Sang et al. 2023). They induced more MPXV‐specific CD8+ effector memory T‐cells (TEM) than CD4+ TEM cells, highlighting a robust memory T‐cell response. Both vaccines protected mice following challenge. Moreover, two multivalent mRNA vaccines were developed: Rmix4, encoding M1, A29, B6 and A35, and Rmix6, which also includes H3 and E8, were produced by an optimised strategy in which DNA templates were combined at the initial stage, allowing generation of multivalent mRNA vaccines through a single‐process approach. These showed protective efficacy comparable to that produced using conventional, more labour‐intensive techniques (Zeng et al. 2023). Both vaccines induced high titres of neutralising antibodies, and the Rmix6 vaccine induced stronger T‐cell responses. All immunised mice survived lethal challenge, compared to 16.7% of controls. Two other multivalent mRNA vaccines, BNT166a and BNT166c, conferred complete protection in mice, achieving 100% survival and undetectable viremia; similar results were observed in NHPs (Zuiani et al. 2024). Consequently, the vaccines progressed into a phase I/II clinical study (NCT05988203). Finally, using an epitope‐enrichment strategy, an 8‐valent vaccine (Mix‐8) encoding A29, M1, B6, A35, H3, A17, A30 and H2, and the 12‐valent vaccine (Mix‐12) encoding the same as Mix‐8 with an additional four antigens (A28, A21, G2 and I2) were developed and showed different levels of neutralising antibodies with partial protection following challenge, indicating that more optimisation is required (Tai et al. 2025).

## 7 mRNA Vaccines Against Bacterial Infections

The global rise of antibiotic‐resistant bacteria is a major public health threat, with an estimated 5 million deaths attributed to antimicrobial resistance (AMR) in 2019 (Murray et al. 2022). According to the 2022 Global Burden of Disease study, six bacterial pathogens including *Escherichia coli, Staphylococcus aureus, Klebsiella pneumoniae, Streptococcus pneumoniae, Acinetobacter baumannii, and Pseudomonas aeruginosa* were identified as responsible for approximately 75% of AMR‐related mortality. These pathogens are also listed in the WHO priority pathogens list (World Health Organization 2024) and include the ESKAPE group, which are multidrug‐resistant bacteria involved in hospital‐acquired infections and their remarkable ability to evade the effects of multiple antimicrobial agents (Rice 2008). Consequently, the need for effective bacterial vaccines is urgent (Micoli et al. 2021). Despite this, mRNA vaccines targeting bacterial pathogens remain limited compared to those targeting viruses because of issues with bacterial mRNA vaccine design, such as larger genome size, higher number of potential targets, the risk of unwanted mammalian glycosylation and potential misfolding of transmembrane domains in bacterial proteins (Khlebnikova et al. 2024). However, the ongoing research shows promising potential, with many candidates showing protective efficacy and strong immunogenicity in preclinical models (Table 2).

**TABLE 2:** Examples of mRNA vaccines against bacterial and parasitic infections.

Infectious agent	Antigen	Type of mRNA^a^	Model	Route; Dose	Immune response	Bioburden/survival	References
Bacterial infections							
*P. aeruginosa*	PcrV and OprF‐I	nm	Mice	5 μg/25 μg, i.m.	NAb, IgG, IFN‐γ, IL‐2 and IL‐4	75% survival in systemic infection; 100% in burn infection; 2–3‐log_10_ reduction	Wang et al. (2023)
*B. burgdorferi*	19ISP	nm	Guinea pigs	50 μg (2.63 μg per antigen), i.d.	N/A	80% of ticks detached	Sajid et al. (2021)
*M. tuberculosis*	ID91	sa	Mice	1 μg, i.m.	IgG, CD4+ and CD8+ T‐cell (IFN‐γ, TNF‐α, and IL‐2)	0.43–0.847‐log_10_ reduction	Larsen et al. (2023)
	p25 epitope of Ag‐85B and C5 peptide from CFP‐10	sa	Mice	5 μg, sc	IgG, CD4+ and CD8+ T‐cell (IFN‐γ, TNF‐α, and IL‐2)	1 log_10_‐reduction	Mishra et al. (2023)
*M. avium*	ID91 proteins: Rv3619, Rv2389, Rv3478 and Rv1886	sa	Mice	1 μg, i.m.	IgG1, IFN‐γ, TNF‐α, and IL‐2	1–1.5‐log_10_ reduction	Rais et al. (2023)
*Y. pestis*	cp‐caf1	nm	Mice	5 μg, i.m.	IgG1	50%–100% survival	Kon et al. (2023)
	F1 and V	sa	Mice	1 and 5 μg, i.m.	NAb, IgG, IFN‐γ	71% survival	Shattock et al. (2023)
*C. difficile*	TcdA/TcdB/PPEP‐1	nm	Mice	1 μg, i.m.	IgG, IgA, CD4+ and CD8+ T‐cells	Undetectable bacteria with 100% survival	Alameh et al. (2024)
*B. pertussis*	PTX‐S1, FHA3, FIMD/2/3, PRN, DT, TT, RTX, TCFA, SPHB1, BRKA	nm	Mice	10 μg, i.m.	IgG, CD4+ T‐cell (IFN‐γ), CD4+ effector memory T cells (TEM) and CD4+ central memory T cells (TCM)	3–5‐log_10_ reduction	Wolf et al. (2024)
	PTX‐S1, FHA3, FIMD/2/3, PRN, DT, TT, RTX, TCFA, SPHB1, BRKA	nm	Rats	10 μg, i.m.	NAb, IgG	Up to 99% reduction	Bitzer et al. (2024)
Parasitic infections							
Malaria	PfCSP	nm	Mice	10 μg/30 μg, i.m.	NAb, IgG1, IgG2a, IFN‐γ, TNF‐α, and IL‐12p70	40%–88% uninfected mice (sterile protection)	Mallory et al. (2021)
	PfCSP and Pfs25	nm	Mice	3–30 μg i.m.	NAb, IgG1, IgG2a CD4+ and CD8 + T‐cell (IFN‐γ, IL‐2, and TNF‐α)	80%–100% survival	Hayashi et al. (2022)
	Pvs25	nm	Mice	10 μg, i.m.	NAb, IgG, CD4+ T‐cell (IFN‐γ, IL‐2)	N/A	Kunkeaw et al. (2023)
	mOVA + αGC/αGCB	nm	Mice	5 μg, i.v.	N/A	80% uninfected mice (sterile protection)	Ganley et al. (2023)
Chagas disease	Tc24‐C4 protein + GLA‐squalene emulsion, Tc24	nm	Mice	10 μg Tc24 mRNA, sc	IgG, IgG1 and IgG2c, IFN‐γ, IL‐2, IL‐4, IL‐6, IL‐10, IL‐22, and TNF‐α	N/A	Poveda et al. (2023)

^a^ Type of mRNA (nucleoside‐modified; self‐amplifying).

*Mycobacterium tuberculosis* has posed challenges in identifying the protective immunity, hindering the progress of vaccine development (Zhuang et al. 2023). A repRNA‐ID91‐NLC was developed and administered as either a homologous or heterologous vaccine to complement protein‐adjuvant vaccine candidates (Larsen et al. 2023). The ID91 protein adjuvanted with GLA‐SE provided a 0.74‐log_10_ reduction in lung bacterial loads, whereas the mRNA vaccine only achieved a 0.3‐log_10_ reduction. A heterologous strategy using the mRNA‐prime, protein‐boost approach was more effective, reducing bacterial loads by 0.85 log_10_. Interestingly, the order of immunisation had an impact on efficacy, with the mRNA‐prime, protein‐boost regimens being more effective in reducing lung burden and inducing more polyfunctional CD4+ T‐cells than protein‐prime and mRNA‐boost. A self‐adjuvating mRNA vaccine SelmRp25‐C5, expressing the p25 epitope from *M. tuberculosis* antigen‐85B and incorporating a TLR‐2 stimulating C5 adjuvant peptide derived from the CFP‐10 protein, showed a better protective efficacy with a 1‐log_10_ reduction in bacterial burden (Mishra et al. 2023). The observed protection correlated with an increase in Ag85B‐p25‐specific CD4+ T‐cells, elevated levels of IFN‐γ and IL‐2‐secreting T‐cells and an increase in CD4+ effector memory T‐cells. Notably, BioNTech SE has started a Phase 1 clinical trial of BNT164a1 and BNT164b1 mRNA vaccine candidates (NCT05547464). A repRNA‐ID91 vaccine was developed against *Mycobacterium avium* ( *M. avium* ) and administered as either a homologous prime‐boost or a heterologous prime‐boost, with repRNA‐ID91 prime followed by a protein ID91 + GLA‐SE boost (Rais et al. 2023). The heterologous vaccine regimen elicited one to two‐fold higher levels of IFN‐γ, TNF‐α, and IL‐2 and a two‐ to three‐log_10_ increase of IgG1 response against both ID91 and Ag85B antigens following the *M. avium* challenge. Although both regimens generated a robust CD4+ Th1 response, only the heterologous regimen significantly increased IL‐2‐producing CD4 + CD44+ cells and induced Th1 cytokine‐expressing (IFN‐γ, IL‐2 and TNF‐α) CD4+ T‐cells. Moreover, it elicited strong CD8+ cytokine responses including IFN‐γ and TNF‐α. Although all prime‐boost regimens offered protection in mice, the heterologous regimen again demonstrated better efficacy than the homologous protein prime/boost regimen, in terms of lung bacterial load and bacterial dissemination into spleen and liver (Rais et al. 2023).

Despite 50 years of effort, no vaccine has received approval for clinical use to date against *Pseudomonas aeruginosa* (Sainz‐Mejías et al. 2020). Two mRNA vaccine candidates encoding PcrV and OprF‐I (OprF and OprI) induced a mixed Th1/Th2 or slightly Th1‐biased immune responses, provided protection and reduced bacterial burden and inflammation in burn and systemic infection models (Wang et al. 2023). The mRNA‐PcrV vaccine elicited significantly stronger antigen‐specific IgG1 and IgG2a, and IFN‐γ, IL‐2 and IL‐4 immune responses than the mRNA‐OprF‐I. The mRNA‐PcrV + mRNA‐OprF‐I vaccine conferred robust protection, with 100% survival in a burn model and 85% in a systemic infection model. In comparison, the multivalent subunit vaccine containing PcrV and OprF‐I achieved up to 70% protection in the burn model and 50% in the systemic infection model. The mRNA‐PcrV vaccine demonstrated a 2–3‐log_10_ reduction in bacterial load in the burn and systemic infections, respectively.

A three‐dose (10 μg) nm‐mRNA‐LNP vaccine encoding SEB, targeting *Staphylococcus aureus* , induced stronger and more durable immune responses than 30 μg of alum‐adjuvanted protein vaccine, evidenced by high SEB‐specific IgG titres (up to 1:20,000) sustained for over 100 days, enhanced dendritic cell activation, and promoted IFN‐γ secretion by CD4+ and CD8+ T‐cells (Luo et al. 2024). The vaccine conferred 100% survival and a 2 to 4‐log_10_ reduction in bacterial load in the liver, lungs, spleen and kidneys.

Furthermore, mRNA vaccines encoding YidR, either alone or with a tPA signal sequence, were developed to protect against *Klebsiella pneumoniae* infection (Huang et al. 2025). Both vaccines induced Th1‐biased humoral and cellular responses, over 80% bacterial killing in OPK assays, sustained CD4+ and CD8+ T‐cell activation and elevated IL‐2 and IFN‐γ levels. The immunised mice showed up to a 4log_10_ reduction in bacterial burden in the lungs, BAL fluid, and heart, with a 60%–80% survival rate.

Although the current acellular vaccines targeting *Bordetella pertussis* (aP) are highly effective in children, their efficacy declines over time because of a Th1/Th2‐skewed response rather than Th1/Th17 (Chasaide and Mills 2020). Several multivalent mRNA vaccines were evaluated, including mRNA‐P‐4, which encodes key virulence factors (PTX‐S1, PRN, FHA3 and FIM2/3), and the more comprehensive mRNA‐DTP‐10, encoding additional antigens such as RTX, SPHB1, TCFA, BRKA and the diphtheria (DIP) and tetanus (TET) toxins (Wolf et al. 2024). Compared with DTaP, immunised mice with mRNA‐DTP‐6 demonstrated higher levels of IFN‐γ‐producing CD4+ T‐cells, as well as an increase in CD4+ TEM and CD4+ central memory T‐cells (TCM), and it induced more CD8+ TEM and TCM cells than DTaP. The vaccines also elicited robust IgG titres, with mRNA‐DTP‐10 reaching the greatest titres. A 3‐log_10_ reduction in bacterial load in the lungs and trachea for mRNA‐P‐6 and a 5‐log_10_ reduction for mRNA‐DTP‐10 were observed. Furthermore, mRNA‐DTP‐10 resulted in an over 99% reduction in bacterial loads in the lungs of immunised rats compared with controls following an aerosol challenge (Bitzer et al. 2024). The mRNA‐immunised mice reduced bacterial burden by 35.4% and 67.2% compared to DTaP‐ and whole‐cell pertussis‐immunised mice, respectively.

An mRNA‐LNP SP‐cp‐caf1, encoding the *Yersinia pestis* F1 capsule antigen, with optimised GC content (66%), showed significantly enhanced immunogenicity compared to the 45% GC content and achieved 50% survival following a lethal challenge (Kon et al. 2023). Two modifications, a human Fc conjugated SP‐cp‐caf1 construct to enhance stability and a signal peptide‐free construct, were also evaluated. The SP‐cp‐caf1‐hFc construct showed 100% survival against a lethal dose challenge after a single dose, whereas the signal peptide‐free construct required three doses to induce 100% survival. A sa‐mRNA vaccine targeting F1 and V conferred 71% survival against *Y. pestis* challenge, compared to 12.5% in the control group (Shattock et al. 2023). Moreover, bacterial clearance from the spleen was observed in most immunised mice, with over 10‐fold increase in anti‐V IgG antibodies and neutralising antibodies.

A multivalent mRNA vaccine encoding domains from TcdA, TcdB, and PPEP‐1, targeting *Clostridioides difficile* (CDI), showed 2 to 4 fold more anti‐toxin IgG responses compared to the alum‐adjuvanted‐subunit vaccine, which was stable for seven weeks and induced antigen‐specific CD4+ and CD8+ T‐cells (Alameh et al. 2024). Moreover, the mRNA vaccine conferred 100% survival against a lethal infection compared to 20% in the control group. Importantly, more than 6 months after immunisation and primary infection, immunised mice that had recovered and cleared *C. difficile* were rechallenged, resulting in 100% survival, highlighting long‐term memory responses and durable protection against CDI.

No vaccines against *Acinetobacter baumannii* of any type have advanced to clinical trials yet (Ma and McClean 2021). Multiepitope mRNA vaccines have been designed, and *in silico* immune simulations revealed that they may induce high IgG responses, IFN‐y and IL‐2 levels (Xu et al. 2024; Ma et al. 2025). However, their immunogenicity and protective efficacy have not been evaluated in animal models to date.

Finally, an nm‐mRNA‐LNP encoding 19 *Ixodes scapularis* proteins (19ISP) was developed to enhance tick bite recognition, reduce engorgement and prevent *Borrelia burgdorferi* infection (Sajid et al. 2021). In guinea pigs, the vaccine triggered early local inflammation at the tick bite site. It disrupted tick feeding, leading to early detachment, lower engorgement weights, and reduced *B. burgdorferi* transmission, showing that 19ISP has potential as either alone or in combination with existing Lyme disease vaccines. Currently, ModernaTX Inc. has started Phase 1/2 clinical trial of two mRNA‐vaccines (mRNA‐1975 and mRNA‐1982) that target Lyme disease (NCT05975099).

## 8 mRNA Vaccines Against Parasitic Infections

The mRNA vaccine platform has also been evaluated for two parasitic infections, malaria and Chagas disease (CD). The current malaria vaccine, RTS, S, is only moderately protective, reducing severity by 30% in children (Zavala 2022). A PfCSP mRNA‐LNP demonstrated high antibody titres in mice that showed 99% inhibition of *Plasmodium falciparum* sporozoite invasion and development in human hepatocytes, along with high IFN‐γ, TNF‐α and IL‐12p70 levels (Mallory et al. 2021). The vaccine resulted in 40% of immunised mice achieving sterile protection, whereas all control mice developed infections with a 3‐week interval immunisation regimen, and the efficacy improved to 88% with a six‐week interval. Moreover, Pfs25 and PfCSP mRNA‐LNP vaccines showed strong antibody responses, with all mice in the PfCSP mRNA‐LNP groups being fully protected, and 80% of the mice in the Pfs25 + PfCSP mRNA‐LNP group achieved complete protection (Hayashi et al. 2022). The Pfs25 mRNA‐LNP vaccine elicited a Th‐1 skewed response, with elevated CD4+ and CD8+ T‐cell activation in mice. Kunkeaw et al. (2023) reported mRNA‐LNP vaccines encoding Pvs25 induced strong antibody responses and demonstrated 100% transmission‐blocking activity (TRA), whereas the subunit vaccine showed only 63% TRA. Given the critical role of liver tissue‐resident memory T‐cells (Trm cells) in controlling liver‐stage malaria, Ganley et al. (2023) optimised an mRNA‐based vaccine to induce Trm cells by incorporating a type I NKT‐cell agonist (αGalCer), resulting in a substantial generation of liver Trm cells and effective protection. An 80% sterile protection was achieved in mice immunised with the αGC_B_‐adjuvanted RPL6 mRNA vaccine encoding the ribosomal subunit protein L6, with no detectable parasitaemia. Recently, BioNTech has started a phase 1 clinical trial for a PfCSP multivalent mRNA vaccine, BNT165e, in healthy volunteers (BioNTech SE 2024).

The development of Chagas disease vaccines is more limited. An mRNA‐LNP encoding Tc24 demonstrated enhanced immunogenicity as a heterologous mRNA‐protein vaccine, with significantly high levels of activated CD4+ and CD8+ T‐cells and a balanced Th1/Th2/Th17 immune response (Poveda et al. 2023). Subsequently, mRNA‐LNP encoding Tc24 and ASP‐2 was evaluated as either a monovalent or bivalent vaccine (Mancino et al. 2024). The bivalent vaccine induced robust immune responses, with sustained inflammatory (IFN‐γ, TNF‐α, IL‐2, IL‐22 and IL‐6) and anti‐inflammatory (IL‐4, IL‐10) cytokine production. It significantly reduced parasite burden and conferred durable protection up to 126 days.

## 9 Optimising mRNA Vaccines: Overcoming Challenges to Enhance Immunogenicity and Delivery

**FIGURE 3:**
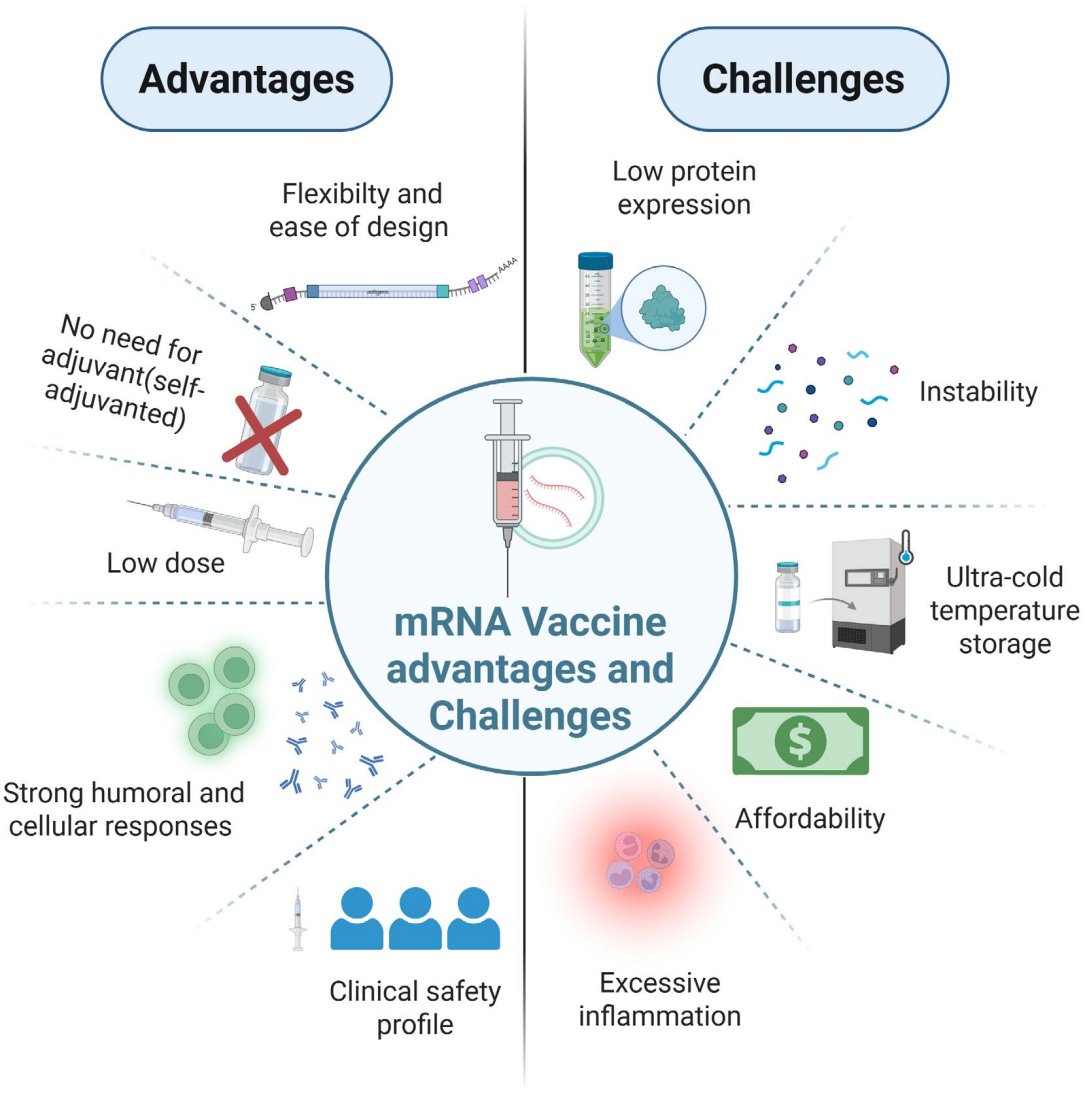
Summary of the advantages and challenges associated with the mRNA vaccine platform. Created with BioRender.com.

Despite the advantages of mRNA vaccines, they still face several challenges, including instability and low protein expression (Jin et al. 2025; Figure 3). For example, the mRNA vaccines can be enhanced by optimising untranslated regions (UTRs), target genes, modifying antigens, and selecting suitable delivery vectors. The 5′‐and 3′‐UTRs regulate mRNA stability, translation efficiency and protein expression (Reshetnikov et al. 2024). In addition, codon optimisation enhances the translation efficiency and protein expression and reduces recognition by pattern recognition receptors (PRRs) (Wei et al. 2025). CureVac developed a sequence‐engineering strategy by altering codon usage to minimise uridine content and increase GC content, enhancing antigen expression, immunogenicity and reducing uridine‐induced inflammation following mRNA delivery (Thess et al. 2015; Rauch et al. 2021). Moreover, antigen modifications, including mutating functional sites, altering secretion potential or modifying protein conformation, can significantly improve immunogenicity and minimise reactogenicity (Freyn et al. 2021). The immunisation with membrane‐bound HA constructs induced more antibody responses than the secreted form, likely because of the enhanced protein stability from its native transmembrane domain and improved antigen presentation. Moreover, altering the catalytic site of NA to reduce enzymatic activity lowered the immunogenicity, despite conferring protection, whereas disruption of M2 ion channel activity enhanced both immunogenicity and protection (Freyn et al. 2021).

The delivery of mRNA vaccines remains a significant challenge because of degradation, limited stability and inefficient cellular uptake (Wadhwa et al. 2020; Litvinova et al. 2023). Although lipid carriers have shown promise, alternative carriers, including liposomes, liposome‐like nanoparticles, solid lipid nanoparticles, lipid‐polymer hybrid nanoparticles, nanoemulsions, exosomes, and lipoprotein particles, are also being evaluated (Hou et al. 2021).

A key limitation of the mRNA vaccine is its requirement for ultra‐cold storage and short shelf‐life, which restricts the supply of the vaccine to low‐income and middle‐income countries (LMICs) (Wouters et al. 2021). However, lyophilisation has been shown to enhance stability significantly, enabling storage of the mRNA vaccines for 3–6 months at room temperature and up to 2 years under refrigeration, without compromising the antigen expression or immunogenicity (Muramatsu et al. 2022; Voigt et al. 2022; Munoz‐Moreno et al. 2025). Indeed, a major shortcoming of mRNA vaccines is access in LMICs. Issues including affordability, manufacturing capacity and market exclusion have been highlighted in a recent WHO report (World Health Organization 2023). The WHO/MPP technology transfer programme, established in 2021, aimed to develop mRNA vaccine manufacturing in LMICs, but second‐generation vaccines, which overcome shelf‐life issues, must be developed to enable better use in LMICs and improve global access to mRNA vaccine technology (Gsell et al. 2023).

## 10 Clinical Progress of mRNA Vaccines: Trials, Safety and Approvals

The rapid development of mRNA vaccines during the COVID‐19 pandemic highlighted their potential for future use. Currently, many mRNA‐based vaccines have advanced to various clinical trial phases (Table 3) and, as expected considering their relatively limited preclinical development, bacterial and parasitic mRNA vaccines are underrepresented in clinical trials relative to viral counterparts. Noteworthily, mRNA‐1345, a vaccine targeting respiratory syncytial virus (RSV), demonstrated high efficacy in Phase 3, with an efficacy of up to 84% in preventing RSV‐associated lower respiratory tract disease with at least two signs. The vaccine also exhibited an acceptable safety profile: 56% of participants showed local adverse events, and 48% experienced systemic adverse events, most of which were mild to moderate in severity and self‐limiting. Serious adverse events occurred in only 2.8% of cases (Falsey et al. 2023). In June 2024, the European Medicines Agency approved mRNA‐1345, commercially known as mRESVIA, for adults aged 60 years and over (European Medicines Agency 2024). This was followed by U.S. Food and Drug Administration (FDA) approval a year later (U.S. Food and Drug Administration 2025). The mRESVIA vaccine represents the first approved mRNA vaccine targeting a viral infection other than SARS‐CoV‐2, marking a significant milestone in the clinical application of mRNA vaccine technology.

**TABLE 3:** Examples of the clinical trials of mRNA vaccines against viral, bacterial and parasitic infections.

Vaccine name	Infection	Antigen	Phase	Population	Vaccine efficacy/protection	Company	References
mRNA‐1010	Influenza	Quadrivalent HA of Influenza A (H1N1, H3N2) & Influenza B (Yamagata Lineage, Victoria Lineage)	phase 3	≥ 18 years	Safe, NAb	ModernaTX Inc.	Lee et al. (2023); Soens et al. (2025)
mRNA‐1011.1, mRNA‐1011.2, and mRNA‐1012.1		Quadrivalent HA & HA sequences of H3N2, and H1N1	Phase 1/2	50–75 years	Safe, NAb	ModernaTX Inc.	Hsu et al. (2025)
Quadrivalent influenza modRNA vaccine (qIRV)		Quadrivalent HA of influenza	Phase 1/2	18–85 years	Safe, NAb and T‐cell responses	Pfizer	Branche et al. (2025)
LNP CL‐0059 and LNP CL‐0137	RSV	2 different LNPs	Phase 1/2	18–50 years and ≥ 60 years	Ongoing	Sanofi	NCT05639894
RSVictory		mRNA‐1345 coadministered with a seasonal influenza vaccine (Afluria Quadrivalent)	Phase 3	≥ 50 years	Ongoing	ModernaTX Inc.	NCT05330975
mRNA‐1345		Stabilised prefusion form of the F (preF) RSV protein	Phase 2–3 trial	≥ 60 year	68%–84% prevention of the infection	ModernaTX Inc.	Wilson et al. (2023)
mRNA‐1325 and mRNA‐1893	Zika Virus	prmE	Phase 1	18–49 years	mRNA‐1893: safe & NAb	ModernaTX Inc.	Essink et al. (2023)
mRNA‐1644 and mRNA‐1644v2‐Core	HIV	eOD‐GT8 60mer and g28v2 60mer	Phase 1	18–50 years	Preliminary data (NAb, IgG)	ModernaTX Inc. and IAVI	NCT05001373
mRNA‐1608	HSV	gC, gD, gE	Phase 1/2	18–55 years	Ongoing	ModernaTX Inc.	NCT06033261
GSK3903133A	Rabies	sa‐mRNA encodes RABV‐G	Phase 1	18–40 years	Ongoing	GSK	NCT04062669
CV7202	Rabies	RABV‐G	Phase 1	18–40 years	Safe &NAb	CureVac	Aldrich et al. (2021)
BNT166	Monkeypox	A35, B6, H3, and M1	Phase 1/2	18–65 years	Ongoing	BioNTech SE	NCT05988203
BNT164a1 and BNT164b1	Tuberculosis	Mtb antigens of undisclosed identity	Phase 1	≥ 18 years	Ongoing	BioNTech SE	NCT05547464
mRNA‐1975 and mRNA‐1982	Lyme disease	OspA SR1‐7	Phase 1/2	18–70 years	Ongoing	ModernaTX Inc.	NCT05975099
BNT165b1	Malaria	PfCSP	Phase 1	18–55 years	Ongoing	BioNTech SE	NCT05581641

Myocarditis and pericarditis have both been associated with mRNA vaccines and are adverse events of special interest in current clinical trials (Alami et al. 2023). Post‐authorisation monitoring of COVID‐19 mRNA vaccines has shown that pericarditis and myocarditis occur at very low frequencies (1 < in 10,000) and mostly in young men (Gargano et al. 2021; Buoninfante et al. 2024). Recently, a Phase 3 trial on a booster SA‐mRNA COVID‐19 vaccine reported one adverse event of special interest for the detection of myocarditis and pericarditis in the SA‐mRNA group compared with three cases in the Comirnaty group (Oda et al. 2024). A more recent study on a next‐generation mRNA‐1283 COVID‐19 vaccine (11,454 participants) showed no pericarditis or myocarditis events, but post‐marketing evaluation is better placed to identify rare events. The recently approved mRESVIA vaccine (mRNA‐1435) reported 2 events of acute pericarditis that were not considered vaccine‐related, and no myocarditis and no safety concerns were evident (Wilson et al. 2023). Finally, another potential safety concern includes a rare instance of +1 ribosomal frameshifting resulting in mRNA mistranslation in recipients of SARS‐CoV‐2 mRNA vaccines; however, no adverse clinical outcomes were observed (Mulroney et al. 2024).

## 11 Conclusions

mRNA vaccines represent a promising platform to target various infectious diseases beyond COVID‐19. They can elicit both CD4+ and CD8+ T‐cell responses; do not require nuclear delivery; have no risk of genomic integration; and offer design flexibility and rapid manufacturing. However, mRNA vaccines targeting bacterial infections remain quite limited compared to those targeting viral infections. Therefore, there is an urgent need to increase the focus on developing novel mRNA vaccines targeting bacterial infections, particularly the antibiotic‐resistant bacteria.

## Author Contributions

**Nouran Rezk:** conceptualization, writing – original draft, writing – review and editing, visualization. **Siobhán McClean:** conceptualization, funding acquisition, writing – review and editing, visualization, supervision.

## Conflicts of Interest

The authors declare no conflicts of interest.

## Data Availability

The authors have nothing to report.
